# WPDSI: A deep learning method for wheat phenology detection from single-temporal images

**DOI:** 10.1016/j.plaphe.2025.100144

**Published:** 2025-12-04

**Authors:** Yan Li, Yucheng Cai, Xuerui Qi, Suyi Liu, Xiangxin Zhuang, Hengbiao Zheng, Yongchao Tian, Yan Zhu, Weixing Cao, Xiaohu Zhang

**Affiliations:** a National Engineering and Technology Center for Information Agriculture, Nanjing Agricultural University, Nanjing, 211800, China; b Key Laboratory for Crop System Analysis and Decision Making, Ministry of Agriculture and Rural Affairs, Nanjing, 211800, China; c Jiangsu Collaborative Innovation Center for Modern Crop Production, Nanjing, 211800, China

**Keywords:** Wheat, Phenology monitoring, Knowledge distillation, Attention transfer

## Abstract

Accurate monitoring of wheat phenology is critical for ensuring wheat production. Recent advances in deep learning have enabled the automated detection of wheat phenology in the field. In particular, deep learning models using multi-temporal image series have addressed the challenge of low accuracy in models that only use spatial features by incorporating dynamic aspects of the wheat growth process. However, utilizing multi-temporal image series introduces challenges such as model parameter redundancy, complex inference processes, and difficulties in real-time deployment. To address these issues, this study presents an optimization method for deriving wheat phenology from single-temporal images (WPDSI) that combines knowledge distillation and multi-layer attention transfer. The proposed approach employs knowledge distillation. In this framework, a teacher model extracts spatiotemporal features from multi-temporal image-series and generates soft labels to guide a student model trained on single-temporal images. This reduces model complexity and input data requirements. Multi-layer attention transfer allows the student model to inherit feature representations from multiple layers of the teacher model. This enhances its ability to capture key phenological characteristics and supports interpretability through attention mechanisms. The proposed method achieves an overall accuracy (OA) of 0.927, comparable to models trained on multi-temporal image series. Furthermore, the model demonstrates strong generalization on unseen datasets, enhancing real-time performance and computational efficiency while maintaining high accuracy, providing a practical solution for deriving wheat phenology in the field. The dataset is available at https://github.com/phenology-detection/WPDSI.

## 1 Introduction

Wheat phenology detection is a fundamental aspect of precision agriculture management, as it provides farmers with crucial insights into the growth stages of their crops. This, in turn, enables more informed decision-making, leading to optimized agricultural operations and ultimately enhancing wheat yield and quality [1]. However, traditional methods rely mainly on manual observation, making them inefficient, costly, and susceptible to subjective bias, leading to significant inaccuracies and compromising the reliability of the results. Moreover, manual observation falls short of meeting the requirements of modern agriculture for large-scale and continuous monitoring [2]. Recent use of vegetation indices (VIs), such as normalized difference vegetation index (NDVI) [3], excess green index (ExG) [4], and green-red vegetation index (GRVI) [5], has provided limited improvements in efficiency and accuracy of phenology detection. Nevertheless, these methodologies encounter difficulties differentiating between phenological stages with close resemblance and depend on expert prior knowledge and substantial time-series data, which restricts their generalizability and widespread adoption.

The advent of deep learning has engendered novel prospects for detecting wheat phenology. Deep learning models can automatically extract key features, such as leaf morphology, stem height, and spike formation, from images, thereby accurately reflecting changes in wheat growth across different phenological stages [6]. Compared with traditional methodologies, deep learning models have been shown to enhance accuracy and automation, demonstrating robust adaptability to variable environmental conditions [[7], [8], [9]]. Furthermore, through large-scale data training, deep learning models undergo continuous optimization. As the volume of data increases, the adaptability of these models to diverse environments and wheat varieties improves, thereby enhancing the precision of phenology detection [10]. However, most existing deep learning models are trained using single-temporal images [11,12]. While single-temporal images can capture spatial structural features of wheat, they cannot account for changes in these structures over time, which hinders accurate differentiation between phenological stages. Some studies have attempted to analyze the relationship between phenology and time by inputting single-temporal images in temporal order. However, they have not fully addressed the inherent correlations between different temporal orders, making it challenging to comprehensively capture the dynamic features of wheat phenology [13,14]. Several models trained on multi-temporal image series datasets have demonstrated encouraging results in monitoring crop phenology at the macro scale using satellite remote sensing. However, the inherently low temporal resolution and delayed data acquisition of satellite imagery make it challenging to support real-time wheat phenology monitoring [[15], [16], [17]]. To overcome these limitations, some studies have proposed the use of wheat phenology detection models based on field-deployed Internet of Things (IoT) systems that collect multi-temporal image series [[18], [19], [20], [55]]. These models capture images of wheat at different stages over time, forming a time-series dataset that enhances the extraction of spatiotemporal features. This approach accurately captures dynamic changes during wheat phenological stages and can improve detection accuracy. Despite their effectiveness, these models rely on complex deep neural network architectures to handle high-dimensional spatiotemporal features and require storing and processing large volumes of sequential image data. Consequently, these models face substantial computational demands, onerous data management workflows, and constrained real-time performance, impeding their practical implementation [21]. This issue is further exacerbated by the prevailing research paradigm in deep learning, which prioritizes large-scale architectures, characterized by increased model parameters, expanded training datasets, and prolonged training cycles, to achieve performance gains [22,23]. This approach, however, significantly increases energy consumption and hardware costs, limiting its deployment on resource-constrained edge devices. Consequently, reducing computational resource requirements without compromising overall accuracy remains a pivotal challenge for effectively implementing wheat phenology detection models.

To address the abovementioned challenges, this study proposes an optimization method for deriving wheat phenology using single-temporal images. This approach integrates knowledge distillation and attention transfer, allowing the model to maintain the time-series feature extraction benefits of the multi-temporal model while reducing input complexity and computational demands. The proposed method utilizes knowledge distillation to enable the single-temporal model to learn dynamic features from the multi-temporal model. Meanwhile, attention transfer directs the model to focus on key feature areas associated with different phenological stages. This enhances our understanding of the model's operational mechanisms and allows for targeted optimization based on the insights gathered. This, in turn, boosts the model's performance and reliability in practical applications. In summary, through knowledge distillation and attention transfer, the proposed optimization method effectively reduces input complexity and computational costs while maintaining high accuracy, offering a practical solution for real-time applications in smart agriculture.

## 2 Materials and methods

### 2.1 Data collection

Two field experiments were conducted in this study during the wheat growing seasons of 2022–2023 and 2023–2024. The experiments took place at the Baima Experimental Station of Nanjing Agricultural University, located in Lishui District, Nanjing, Jiangsu Province, China (119°09′ E, 31°37′ N). The experimental designs are outlined below:

Experiment 1. This experiment involved a single variety (Nannong 1632), with six plots, each measuring 90 ​m^2^ (30 ​m ​× ​3 ​m). The sowing method used was manual broadcast seeding.

Experiment 2. This experiment incorporated two varieties (Zhenmai 168 and Nongmai 88), two planting density levels (2.4 million plants/ha and 1.5 million plants/ha), and three nitrogen levels (90, 180, and 270 ​kg/ha), with a total of 12 plots, each measuring 36 ​m^2^ (6 ​m ​× ​6 ​m). The sowing method used was manual row seeding.

**Fig. 1:**
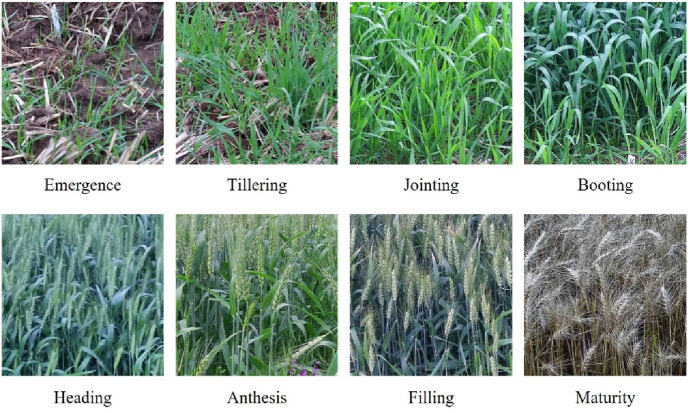
Digital color images of the part of the wheat field in each phenology.

An RGB camera was employed to capture wheat images. The camera model was Hikvision E DS-2DE4223IW-D/GLT/XM (Hangzhou Hikvision Digital Technology Co., Ltd., China). Image resolution was 1920 ​× ​1080 pixels. Images were collected daily from 8:00 to 17:00. The camera was mounted at a height of 3 ​m above ground level. The spatial resolution was 0.05 cm/pixel. The vertical viewing angle ranged from −20° to −80°, which was manually adjusted for each plot. Fig. 1 shows the growth of wheat at each stage. Images collected at different timestamps within a day were labeled as belonging to the same phenological stage. Concurrently, the onset dates of all phenological stages, from emergence to maturity, were recorded according to the standard definitions of wheat growth stages [24].

A two-year field experiment on wheat was conducted to establish a robust data foundation for model training and evaluation. Data collected during the first year were utilized for model training, validation, and testing. Following our previous study, the images were preprocessed to construct a standardized image series dataset [18]. Original images were cropped to 1000 ​× ​1000 pixels and manually labeled with phenological stage. We then organized the images into time-series samples, with each sample comprising 30 temporally ordered images to capture phenological dynamics. Each 30-image series was treated as an independent sample, and the dataset was subsequently split at the series level—rather than at the single-image level—into training, validation, and test sets in a 6:2:2 ratio. Data augmentation techniques, including brightness adjustment, flipping, and random rotation, were applied to the training image-series samples, resulting in 13,648 multi-temporal image-series samples (Table 1). The second-year dataset was used as an unseen test set to assess model generalization. Unlike the first-year dataset, the second-year dataset consisted of single-temporal images as opposed to multi-temporal image series, thereby obviating the necessity for constructing time-series datasets. The second-year study amassed a total of 25,002 wheat images.

**Table 1:** The number of image-series samples of different wheat phenological stages.

Phenological stage	Training set (Before augmentation)	Training set (After augmentation)	Validation set	Test set	Overall (After augmentation)
Emergence	612	1836	204	204	2244
Tillering	716	2148	254	254	2656
Jointing	490	1470	163	163	1796
Booting	388	1164	112	112	1388
Heading	370	1110	121	121	1352
Anthesis	284	852	98	98	1048
Filling	366	1098	105	105	1308
Maturity	496	1488	184	184	1856

ALL	3722	11166	1241	1241	13648

### 2.2 Methods

**Fig. 2:**
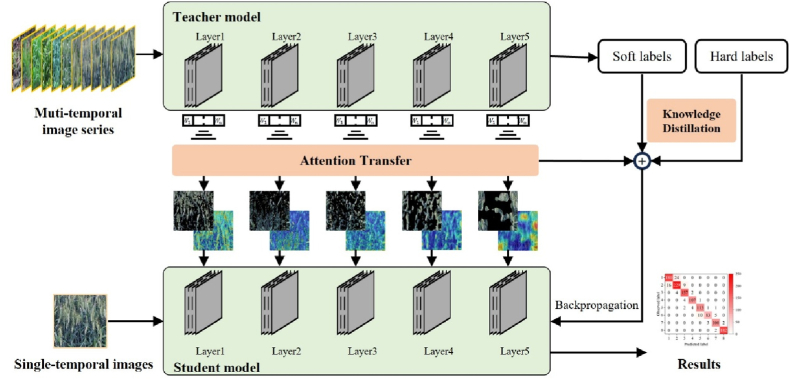
Technical framework.

This study proposes a multi-layer feature fusion optimization strategy based on knowledge distillation [25] and attention transfer [26] to optimize the wheat phenology detection model using single-temporal images (Fig. 2).

#### 2.2.1 Knowledge distillation

Knowledge distillation is a common approach in deep learning, particularly in the context of model compression and performance enhancement. Knowledge distillation is based on the "teacher-student" training paradigm, where both networks are built upon a ResNet-50 backbone in this study. In the proposed framework, the teacher model consists of a ResNet-50 feature extractor followed by an LSTM module, with the convolutional network responsible for spatial feature extraction and the recurrent module for temporal memory. The student model adopts a simplified architecture using only the ResNet-50 backbone. Knowledge is transferred from a complex, high-capacity teacher model to a lightweight student model. This allows the student model to achieve high performance with a simplified architecture. In this study, the teacher model was trained on a multi-temporal image series dataset of wheat phenology to capture spatiotemporal features across different growth stages. After extensive training, the teacher model demonstrated the capability to extract high-level features and generate soft labels, defined as probabilistic distributions over all classes for each input sample. These soft labels assign the highest probability to the correct class and contain implicit information, such as uncertainty at phenological stage boundaries and similarities between adjacent stages. In contrast, hard labels refer to the manually annotated one-hot vectors in the dataset, where the correct class is assigned a value of 1, and all others are set to 0. During training, the student model learns both from hard labels for phenological stage classification and from soft labels generated by the teacher model. This helps the student model capture subtle patterns and inter-class relationships. The temperature parameter (T) is a pivotal hyperparameter in knowledge distillation, employed to modify the output distribution from the softmax layer. The output probabilities are softened by increasing the temperature, thereby making the influence of non-target classes more prominent (Formula 1).

(1) $$q_{i}=\frac{\exp\left(z_{i}/T\right)}{\sum_{j=1}^{N}\;\exp\left(z_{j}/T\right)}$$

where $q_{i}$ represents the output probability for each class, $z_{i}$ represents the logit for each class, N represents the number of classes. T represents the temperature parameter. When *T* ​= ​1, the standard softmax function is utilized. T was set to 3 in this study, following common practice in knowledge distillation [25].

The loss function is defined as the weighted combination of the loss from soft labels and the loss from hard labels. The class probability distribution by the teacher model at temperature *T* serves as the soft label. The first part of the loss function is the cross-entropy between the student model's softmax output at temperature *T* and the soft label, while the second part is the cross-entropy between the softmax output at *T* ​= ​1 and the hard label (Formula 2).

$$L=\alpha L_{soft}+\left(1-\alpha \right)L_{hard}$$

(2) $$\begin{array}{l} p_{i}^{T}=\frac{\exp\left(v_{i}/T\right)}{\sum_{j}^{N}\;\exp\left(v_{j}/T\right)}\\ q_{i}^{T}=\frac{\exp\left(z_{i}/T\right)}{\sum_{j}^{N}\;\exp\left(z_{j}/T\right)}\\ L_{soft}=-\sum_{i}^{N}p_{i}^{T}\;\log\left(q_{i}^{T}\right) \end{array}$$

$$\begin{array}{l} q_{i}^{1}=\frac{\exp\left(z_{i}\right)}{\sum_{j}^{N}\;\exp\left(z_{j}\right)}\\ L_{hard}=-\sum_{i}^{N}c_{i}\;\log\left(q_{i}^{1}\right) \end{array}$$

where $p_{i}^{T}$ and $q_{i}^{T}$ denote the softmax output of the teacher and the student model for class *i* at temperature *T*, respectively. $v_{i}$ and $z_{i}$ denote their logits. *N* is the number of classes. $c_{i}$ is the ground truth for class *i*, where $c_{i}\in \left\{0,1\right\}$.

#### 2.2.2 Attention transfer

In this study, a multi-layer feature fusion strategy based on attention transfer was employed to enhance the student model's performance. Specifically, attention maps from various layers of the teacher model were transferred to the student model, enabling it to focus on critical image regions even under resource-constrained conditions, thereby improving the accuracy of phenological stage detection. The approach commenced with the computation of attention maps at each layer of the teacher model. These maps reflect the spatial focus of the teacher model across different feature levels when processing input images. These maps were then employed as supervisory signals to guide the student model to concentrate on significant regions during feature extraction. Combined with knowledge distillation, the student model learned the final output distributions from the teacher model and acquired intermediate-level features by mimicking the teacher's internal representations. This enhanced the student model's capacity to discern salient features at various depths within the network. The L2 norm was utilized as a distance metric to quantify the similarity between the attention maps of the teacher and student models. The final loss function integrated the distillation and attention transfer loss via a weighted combination. The distillation loss function guided the student model towards approximating the teacher model's output distribution. In contrast, the attention transfer loss encouraged the alignment of intermediate feature representations between the two models. This training strategy enabled the student model to maintain strong global feature extraction capabilities while accurately capturing fine-grained, phenology-relevant information in localized image regions (Formula 3).

(3) $$\begin{array}{l} L_{attention}={\left\|A_{S}^{i}-A_{T}^{i}\right\|}_{2}\\ L=\alpha L_{soft}+\left(1-\alpha \right)L_{hard}+\beta L_{attention} \end{array}$$

where $A_{S}^{i}$ and $A_{T}^{i}$ represent the attention maps of the student and teacher models at the *i*-th layer. The hyperparameters α and β were set to 0.7 and 0.001, respectively, to follow prior work on combining cross-entropy, distillation, and attention transfer losses [25,27].In classification tasks, higher activation values of neurons indicate greater importance and attention. Consequently, visualizing these maps facilitates researchers in analyzing the model's optimization strategy from the perspective of interpretability. In this study, the attention maps from each intermediate layer were flattened along the channel dimension and visualized alongside the original image, thereby providing insights into the model's focus during different phenological stages.

## 3 Experiment and results

### 3.1 Experimental parameter settings

Experiments were conducted on a server configured with dual Intel® Xeon® CPUs, seven NVIDIA® TESLA® A100 GPUs (40 ​GB), 1 ​TB of RAM, and running Ubuntu 20.04. The backpropagation algorithm [28] was utilized for parameter optimization of the deep learning model. The Adam optimizer was selected as the optimization algorithm, which facilitates a balance between model performance and the convergence velocity [29]. Moreover, dropout was employed to prevent overfitting [30]. This approach applies dropout regularization by randomly deactivating outputs of specific hidden neurons, thereby reducing model complexity and enhancing generalization. During training, the hyperparameters batch size, learning rate, and dropout rate were set as 16, 0.0001, and 0.3, respectively.

### 3.2 Performance evaluation

To objectively and efficiently evaluate model performance, this study employed five evaluation metrics: confusion matrix, overall accuracy (OA), F1-score (F1), kappa coefficient (Kappa), and mean absolute error (MAE). The confusion matrix, a square N ​× ​N grid, illustrates the relationship between predicted and true labels, allowing for the assessment of classification performance across categories. OA, F1, and Kappa values range from 0 to 1, where higher values indicate superior performance. A sample correctly classified as stage *i* was counted as true positive (TP); if misclassified into another stage, it was false negative (FN); if a sample from another stage was incorrectly predicted as stage *i*, it was false positive (FP); and if a sample from another stage was correctly predicted as a stage different from *i*, it was true negative (TN).

(4) $$OA=\frac{TP+TN}{TP+TN+FP+FN}$$

(5) $$Precision_{i}=\frac{TP_{i}}{TP_{i}+FP_{i}}$$

(6) $$Recall_{i}=\frac{TP_{i}}{TP_{i}+FN_{i}}$$

(7) $$F1\;score_{i}=\frac{2*Precision_{i}*Recall_{i}}{Precision_{i}+Recall_{i}}$$

(8) $$\begin{array}{l} p_{0}=OA\\ p_{e}=\frac{\left(\text{TP}+\text{FP}\right)*\left(\text{TP}+\text{FN}\right)+\left(\text{FP}+\text{TN}\right)*\left(\text{FN}+\text{TN}\right)}{{\left(\text{TP}+\text{TN}+\text{FP}+\text{FN}\right)}^{2}}\\ Kappa=\frac{p_{0}-p_{e}}{1-p_{e}} \end{array}$$

MAE, defined as the average absolute difference between predicted and true labels, quantifies the overall prediction error.

(9) $$MAE=\frac{1}{n}\sum_{i=1}^{n}\left|\hat{y}-y\right|$$

where *i* is the *i*-th class, *n* is the total number of samples. *ŷ* and *y* are the predicted and observed labels, respectively.

### 3.3 Results

**Fig. 3:**
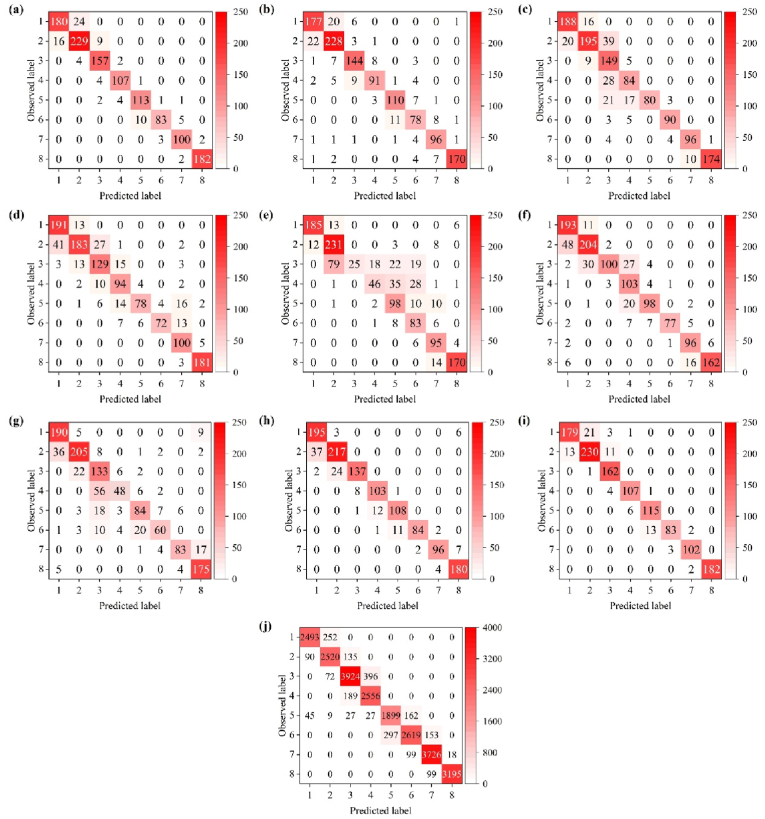
The confusion matrix yielded by eight methods: Proposed (a), ResNet50 (b), MobileNetV3 (c), EfficientNetV2 (d), RepVGG (e), SCNet (f), STViT (g), PhenoNet (h), Teacher model (i), and the proposed method performance on the unseen dataset (j). Serial numbers 1–8 represent the phenological stages: emergence, tillering, jointing, booting, heading, anthesis, filling, and maturity, respectively.

**Fig. 4:**
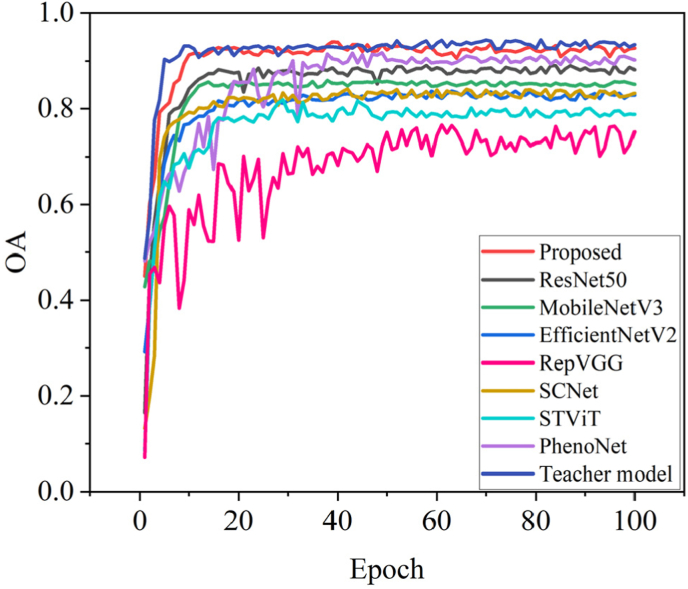
The curves of OA of different methods.

Experimental results demonstrate that the proposed multi-layer feature fusion optimization strategy, incorporating knowledge distillation and attention transfer, effectively improves the accuracy of wheat phenology detection from single-temporal images. The proposed method achieved an OA of 0.927, a MAE of 0.075, an F1-score of 0.929, and a Kappa coefficient of 0.916. In addition, the proposed method is compared with other image classification algorithms, including ResNet50 [31], MobileNetV3 [32], EfficientNetV2 [33], RepVGG [34], SCNet [54], STViT [35], and PhenoNet [19]. All models used for comparison were trained with identical data splits, preprocessing, and augmentation strategies. The pre-trained weights of these models were utilized. The proposed method achieves overall accuracy improvements of 4.5 ​%, 7.6 ​%, 9.9 ​%, 17.5 ​%, 9.5 ​%, 13.9 ​%, and 2.5 ​%, respectively. Notably, the proposed lightweight student model achieved performance almost equivalent to that of the complex teacher model, with a mere 0.8 ​% reduction in overall accuracy. On the test set, the proposed model demonstrated the most prominent diagonal distribution across eight reproductive stages, with a significant decrease in misclassification (Fig. 3). For the middle stages, such as jointing (3), booting (4), anthesis (5), and flowering (6), all models showed obvious confusion. These stages involve continuous and indistinguishable changes in plant morphology: canopy height gradually increases, leaf number and angle change gradually, and the spike is not yet fully exposed. There is a lack of clear visual mutations in the RGB images, making it difficult for the models to accurately distinguish. The proposed model significantly improved discrimination in these critical, most challenging stages, indicating its stronger ability to extract fine-grained reproductive-stage features. To further evaluate the model's generalization ability, the optimized student model was tested on an unseen wheat dataset collected in the second year. The outcomes demonstrated the model's capacity for effective generalization, as evidenced by an overall accuracy (OA) of 0.917, a mean absolute error (MAE) of 0.090, an F1-score of 0.914, and a kappa coefficient of 0.905. The confusion matrix on the unseen data set shows that the proposed method maintains a clear diagonal distribution, verifying its good generalization ability across different lighting conditions, variety differences, and scene changes (Table 2, Fig. 3, Fig. 4).

**Table 2:** The quantitative comparison of different methods. The asterisk represents the performance on the unseen dataset.

Method	OA	MAE	F1	Kappa
Proposed	0.927	0.075	0.929	0.916
ResNet50	0.882	0.173	0.876	0.862
MobileNetV3	0.851	0.185	0.852	0.827
EfficientNetV2	0.828	0.222	0.821	0.801
RepVGG	0.752	0.394	0.699	0.711
SCNet	0.832	0.227	0.832	0.805
STViT	0.788	0.352	0.762	0.753
PhenoNet	0.902	0.130	0.905	0.887
Teacher model	0.935	0.069	0.936	0.924

Proposed∗	0.917	0.090	0.914	0.905

## 4 Discussion

The quality of the input data is critical to the performance of models used to detect wheat phenology. Whilst multi-temporal image series have been shown to provide superior overall accuracy compared to single-temporal image inputs, improving models based on single-temporal data can significantly enhance their practicality and deployment flexibility. In deep learning models, the quality of the input data has been shown to impact the model's performance directly [36]. Research in wheat phenology detection can be categorized into two types: those based on single-temporal images and those based on multi-temporal image series. Single-temporal images contain only spatial structural features and lack temporal sequence features, making it difficult to fully capture the dynamic growth process of wheat, resulting in relatively lower overall accuracy. Conversely, multi-temporal image series have been shown to address this limitation by incorporating temporal dimension information [56]. Previous studies have demonstrated that integrating spatiotemporal features into the model architecture can significantly improve wheat phenology detection accuracy [18]. However, input based on the multi-temporal image series has limitations in practical applications and deployment. For instance, storing early-stage images becomes challenging in the later stages of wheat phenology, and the presence of missing images within the sequence can substantially affect the performance. The teacher model that relies on multi-temporal image series requires matching across consecutive frames, resulting in a parameter count of 33.88 million, representing a 28.3 ​% increase over the student model, thereby incurring greater computational and storage overhead. During the training phase, the student model requires 1.1 ​h, whereas the teacher model requires 7.45 ​h due to the extraction and learning of complex spatiotemporal features. Regarding inference efficiency, the floating-point operations (FLOPs) of the student model are 21.59 GFLOPs, and it takes only 0.04 ​s to process each image, achieving an inference speed of 25 frames per second (FPS), which meets the requirements for real-time detection. The teacher model has 1446.66 GFLOPs and takes an average of 1.76 ​s to process a single image series, significantly reducing its real-time performance. The proposed model in this study significantly reduces the time and cost for model development and optimization (Table 3). Consequently, developing a wheat phenology detection model applicable to the single-temporal image while effectively integrating spatiotemporal features is imperative. This research direction enhances the accuracy and improves the model's applicability in agricultural production. The model is designed to meet the requirements of smallholder farmers, who require real-time monitoring under limited resources.

**Table 3:** Comparison of the performance of the student and the teacher model.

Method	Input Requirement	OA	Parameters (M)	Inference Speed (s)	FPS	Training Time (h)	FLOPs (GFLOPs)
Student model	Single image	0.927	26.40	0.04	25.00	1.10	21.59
Teacher model	Image series	0.935	33.88	1.76	0.57	7.45	1446.66

**Fig. 5:**
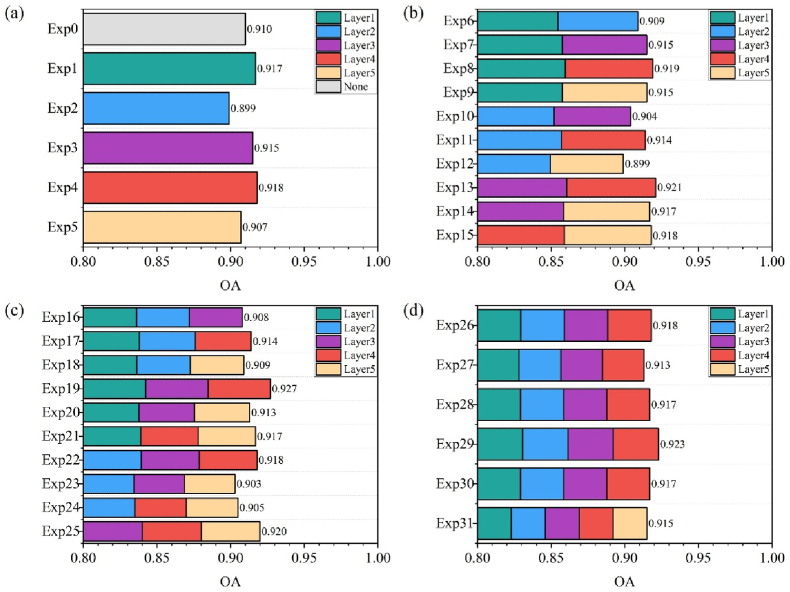
Results based on multi-layer attention transfer. (a) knowledge distillation and attention transfer by one layer, (b) attention transfer by two layers' fusion, (c) attention transfer by three layers' fusion, (d) attention transfer by four or five layers' fusion.

A knowledge distillation and attention transfer based on the multi-layer feature fusion strategy has been shown to significantly enhance the accuracy of models based on single-temporal image inputs. The proposed method retains the spatiotemporal feature fusion capabilities of multi-temporal image series datasets while demonstrating the convenience and real-time applicability of the single-temporal image in practical scenarios. The application of knowledge distillation enables the accuracy of the single-temporal image model to approach that of complex models based on multi-temporal image series, thereby greatly enhancing its applicability [37]. Previous studies have relied on large-scale multi-temporal image series to extract dynamic features unsuitable for application scenarios with limited computational resources. This study proposes a novel approach to knowledge distillation, whereby the spatiotemporal features embedded in a complex multi-temporal image series model (the teacher model) are transferred to a single-temporal image model (the student model) through soft labels [38]. This process enables the student model to acquire the intricate characteristics of diverse phenological stages and discern their indistinct boundaries through soft labels [39], facilitating effective wheat phenology detection under single-temporal image conditions. Additionally, attention transfer enhances model accuracy by utilizing multi-layer feature combinations [40]. In wheat phenology detection, it is imperative to acknowledge that distinct growth stages are associated with disparate feature levels within the model. A myopic reliance on a solitary feature level is often inadequate in comprehensively capturing wheat growth status. The introduction of attention transfer effectively addresses this issue by extracting important attention regions from each layer of the teacher model and transferring them to the student model, enabling the student model to better focus on key feature regions within the images [27,41,42]. To analyze the effect of attention transfer at different layers, an ablation study was conducted by incorporating the attention transfer mechanism into five layers of the model and comparing the performance of single-layer transfer with multi-layer combination transfer. The results show that applying knowledge distillation alone achieves an accuracy of 0.910. The best performance is achieved by combining attention features from the 1st, third, and fourth layers, with an accuracy of 0.927. In contrast, the combination of attention features from the second and fifth layers give the lowest performance, with an accuracy of only 0.899 (Fig. 5). This outcome signifies that the integration of edge and texture information from shallow layers with high-level semantic information from deeper layers optimizes the feature representation capability [43].

**Fig. 6:**
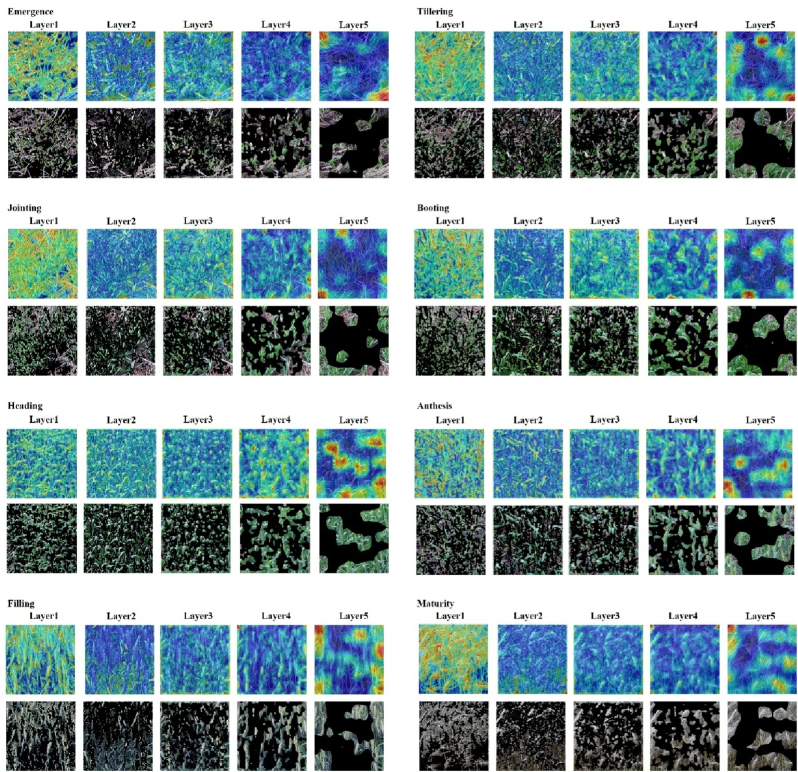
Attention maps of the different layers.

Attention transfer from different network layers contributes unequally to the model's performance, and integrating both shallow and deep features has been shown to enhance the accuracy significantly. The semantic information extracted by deep learning models at varying levels is non-uniform. Shallow features primarily represent low-level characteristics, such as edges and textures [44], while deep features capture higher-level semantic information, such as leaf shapes and spike structures [45]. The features extracted from a single layer are insufficient to comprehensively represent the growth status of wheat, whereas the fusion of multi-layer features contributes to improving the accuracy. This study visualized the attention feature maps at differing model layers, thereby providing an intuitive representation of the feature extraction process for wheat phenology detection. The analysis of feature maps has been found to provide substantial support for enhancing the interpretability of models, thereby helping to reveal the model's focus on features at different phenology stages [[46], [47], [48]]. The model exhibits distinct attention patterns across different layers during the processing of input images. The attention maps at each layer reveal the model's focus during feature extraction across various stages (Fig. 6). The primary focus of the first layer is on low-level features of wheat, including leaf edges and textures during the seedling, tillering, and jointing stages, as well as the edge features of wheat spikes after heading. This observation suggests shallow feature extraction is crucial in distinguishing early growth stages. The second layer, by contrast, is predominantly focused on the soil background. However, given the stability of the background across different growth stages, the transfer of attention at this layer does not yield substantial enhancement of model performance and, in fact, reduces accuracy to 0.899. This finding suggests that, during the training phase, the focus should be directed toward the wheat plants, while the soil background should be given minimal attention. The third and fourth layers progressively shift towards extracting higher-level features, particularly the morphological characteristics of wheat spikes. Following the heading stage, the model's attention concentrates on regions of spike variation, enabling effective differentiation between the heading, anthesis, filling, and maturity stages. This layer-wise shift in attention validates the effectiveness of high-level feature extraction for identifying later growth stages. The fifth and final layer focuses on the entire target region, representing the input image globally.

The proposed strategy integrates knowledge distillation and attention transfer, allowing the student model to learn the attention patterns of the teacher model across intermediate layers. This enhances feature extraction and improves the accuracy of phenological stage detection. This multi-layer integration enables the model to adjust its attention focus dynamically and effectively capture phenology-relevant features [49]. Quantitative contributions of knowledge distillation and attention transfer were analyzed through ablation (Table 4). Knowledge distillation transfers spatiotemporal features from the complex multi-temporal image series teacher model to the single-temporal image student model. This enables the student to learn complex features across different phenological stages and accurately detect wheat phenology from single-time images. For attention transfer, attention maps are first extracted from each teacher layer to identify key focus areas. These maps are then transferred to the student model, helping it focus on key image regions and further enhancing the accuracy of the model based on single-temporal image inputs. Since the attention transfer in this study was based on knowledge distillation, there was no separate attention transfer. A dedicated ablation analysis of attention transfer at different layers is presented in Fig. 5. Future research may focus on tailoring hierarchical feature extraction strategies for specific phenological stages to further enhance detection performance and model generalization.

**Table 4:** The ablation analysis results of WPDSI.

Knowledge distillation	Attention transfer	OA	MAE	F1	Kappa
		0.882	0.173	0.876	0.862
✓		0.910	0.120	0.908	0.895
✓	✓	0.927	0.075	0.929	0.916

The single-temporal image model offers real-time capability and deployment flexibility, rendering it suitable for a wide range of agricultural scenarios and providing farmers with efficient and accurate phenological monitoring tools. The model exhibits significant benefits for deployment in practical agricultural settings, as it requires only a single image for inference and does not rely on any auxiliary data. This contrasts with complex teacher models based on multi-temporal image series, which require continuous input of images. This dependency increases the difficulty of data acquisition and imposes considerable computational and storage costs during deployment, thus limiting their broader applicability. In large-scale farming environments, real-time crop growth monitoring is imperative for enhancing management efficiency and crop yields [50,51]. The improved wheat phenology detection model developed in this study is adaptable to various agricultural scenarios. It can accurately identify growth stages in open-field environments without relying on additional information such as meteorological data. This enhanced flexibility, real-time performance, and user-friendliness broaden the model's applicability, thus offering farmers a more practical monitoring solution. The model's capacity for rapid deployment and real-time feedback enables practical support for farmers in the timely management of adjustments, thereby contributing to enhanced wheat yield and quality. The proposed single-temporal image model offers real-time performance and deployment flexibility. However, its training process still relies on a comprehensive image dataset that covers the entire wheat phenological stage. Specifically, the training pipeline involves the initial collection of a standard image series dataset representing all growth stages, which is then used to train the teacher model. Subsequently, the student model is trained under the guidance of knowledge distillation. However, the current model is not sufficiently adaptable and may encounter limitations in complex field conditions. Challenges such as pest and disease infestations, weed interference, and crop lodging can compromise the accuracy and hinder generalization in real-world applications [52,53]. To address these issues, future applications should incorporate dynamic feedback and active learning mechanisms that iteratively expand the training dataset and refine the model, thereby enhancing its robustness and adaptability to new regions and crop varieties. Moreover, because our experiments were conducted at a single site, future work will validate the model on multi-site datasets and employ domain-adaptation strategies or transfer-learning to mitigate differences in soil background, illumination, and climate, ensuring reliable phenological monitoring across diverse environments.

## 5 Conclusion

This study proposes a wheat phenology detection model based on single-temporal image inputs (WPDSI), incorporating knowledge distillation and attention transfer. The student model achieves light-weighting and efficient deployment while maintaining comparable performance to the teacher model. This innovative method allows the single-temporal image model to approach the accuracy of complex teacher models while significantly reducing computational resource demands and model storage requirements, providing a new technological pathway for intelligent monitoring and automated management in the agricultural field. The analysis of feature map visualizations provides insights into the contributions of different phenological stages across the model's various layers, offering novel perspectives on understanding the internal mechanisms of the model. Additionally, the findings have considerable potential for application in real-world scenarios, as the model is adaptable to various agricultural environments, including field agriculture and greenhouse farming. The model's deployment flexibility and efficiency make it particularly valuable for monitoring crop growth. The potential for expanding this approach to encompass other crops is significant, with the prospect of comprehensive coverage in intelligent agricultural systems, thereby enhancing crop yields and quality, and promoting the modernization of agriculture.

## Author contributions

Conceptualization: Y.L. and X.Z. Methodology: Y.L. and Y.C. Validation: X.Q. and S.L. Data curation: Y.C. and X.Z. Formal analysis: X.Z. Writing—original draft: Y.L., Y.C., X.Q., S.L., and X.Z. Writing—review and editing: X.Z. Supervision: Y.T. and Y.Z. Project administration: W.C. and Funding acquisition: X.Z. and H.Z. All authors contributed equally to the writing of the manuscript.

## Funding

This research was supported by the National Key Research and Development Program of China (2024YFD2301100), the National Natural Science Foundation of China (Grant No. 32171892), the Qing Lan Project of Jiangsu Universities, and Jiangsu Agricultural Science and Technology Innovation Fund (CX (21) 1006).

## Data availability

The dataset is publicly available at https://github.com/phenology-detection/WPDSI.

## Declaration of interest statement

The authors declare that they have no known competing financial interests or personal relationships that could have appeared to influence the work reported in this paper.
